# Methods for studying group loans, join responsibility, and women's empowerment

**DOI:** 10.1016/j.mex.2022.101749

**Published:** 2022-06-02

**Authors:** Efa Wahyu Prastyaningtyas, Sri Umi Mintarti Widjaja, Hari Wahyono, Endang Sri Andayani, Jack Febrian Rusdi

**Affiliations:** a Fakultas Ekonomi Universitas Negeri Malang, Malang, Indonesia; b Universitas Nusantara PGRI Kediri, Kediri, Indonesia; c Sekolah Tinggi Teknologi Bandung, Bandung, Indonesia

**Keywords:** Debt financing, Loan debt, Household income, Microfinance institution

## Abstract

World researchers have studied various phenomena of loans provided through financial institutions, including a loan strategy with Group Loans. Group Loans are money loans made by a group of people to a microfinance institution. Based on a case study in Nganjuk, East Java, Indonesia, we investigated the phenomena of Group Loans. Group Loans customers in the community are primarily women, and they are generally housewives without having a fixed income unless they receive a stipend from their husbands. Group Loan facilities are provided for women's empowerment, primarily to support productive activities. Unfortunately, these group loans potentially experience problems, especially interms of loan payments. Group members who are not paying debts, wasting money, and borrowing from numerous financial institutions to pay off prior obligations have all been recognised as issues. This study examined methods to anticipate and minimize the risk of Group Loans through the implementation of women's empowerment activities. The proposed method in this study is deemed effective to assist women in avoiding debt owing to Group Loans. This study is expected to be one of the solutions to enhancing women's roles, particularly in minimising the risk of debt through productive activities.

• Discussion of methods to examine the phenomena and problems of Group Loans.

• Discussion of approach methods to credit agency customers who borrow money in groups.

• Methods of exercising women's empowerment in anticipation of Group Loans.

## Graphical abstract

**Figure:**
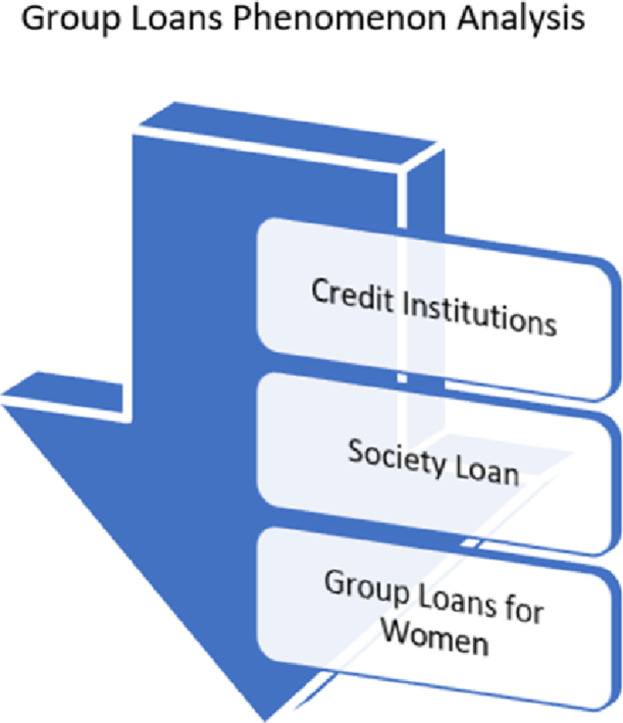

**SPECIFICATIONS TABLE**

**Table:** 

Subject Area;	Economics and Finance
More specific subject area;	*Group Loans*
Method name;	*Methods for Studying Group Loans, Join Responsibility, and Women's Empowerment*
Name and reference of original method;	*N.A.*
Resource availability;	*Rusdi, Jack Febrian (2022), “Dataset of Group Loans in Nganjuk East Java”, Mendeley Data, V1, doi: 10.17632/7gh2vv2rjs.1*[1]

## Introduction

Loans are one option for dealing with financial difficulties. Some experts are studying the phenomenon of loans. Various loan implementations, including the practise of group loans, follow distinct patterns. The community as a whole lends money to credit institutions through group loans.The money is distributed according to the portion agreed by fellow members of the group that owns it [2].

The credit institutions referred to in this study are microfinance institutions, sometimes called joint and multiple liabilities. [3]. Based on the case raised in this study, Group Loans are local wisdom, especially of the Javanese in Indonesia. The implementation of Group Loans is one of the strategies for financial institutions to minimize the risk of bad payments on money lent to the community, considering that the person in charge of the use of funds and payments are made jointly by those who join the community groups [4]. Credit institutions provide convenience through Group Loans [5], primarily to support productive activities for women [6] and to minimize the business risks of credit institutions [2]. Furthermore, Group Loans have a variety of effects on the community, notably on rural families [5].

On the other hand, the loan facility provided by microfinance gives problems to women, especially the high number of debt compared to their income. Other issues that also arise include using credit facilities to pay off debts [7]. Debt problems in women and the role of women need to be studied in more depth to find solutions facing society. For this reason, through this research, we examine alternative steps in overcoming the problem of loans to women, which are carried out through women's empowerment.

This study specializes in the study of methods. The methods discussed are specially to answer research questions, namely how to minimize the risk of payment to Group Loans through implementing women's empowerment activities. The study is based on a community case study at the research site. This study was appointed based on case studies in rural areas in Indonesia, considering that various loan problems occur in rural Indonesia [8].

*The research results that we have done can serve as a basis for other researchers and society, especially in minimizing the risk of debt to the community through empowering women. The method implemented through this study has proven that empowering women can reduce the loan risk for women .*

## Method detail

**Fig. 1:**
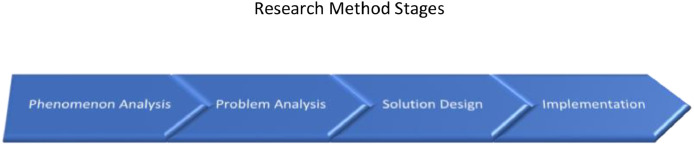
Stages of Group Loans Research and Empowerment of Women

In reviewing this Group Loans, Join Responsibility, and Women's Empowerment methodology, we conducted a study in stages. The study was divided into four parts. These four parts were executed sequentially; phenomenon analysis, problem analysis, solution design, and implementation. Fig. 1 illustrates the method stages, followed by sections detailing the stages.

### Phenomenon analysis

The Group Loans and Women Empowerment phenomenon was analyzed in the first stage.It was conducted inductively [9] where literature review, observations, and interviews were involved. Literature studies used the outcomes of English-language publications drawn from reliable scientific journals and peer-reviewed papers as the foundation of their knowledge. The majority of the scientific studies used come from the Science Direct collection. Simultaneously, the observation was directed to the field in order to analyse the community's conditions in more depth.The next step was the interview method. The Interview sessions were conducted with relevant parties, including financial institutions, community groups, and individuals.

**Fig. 2:**
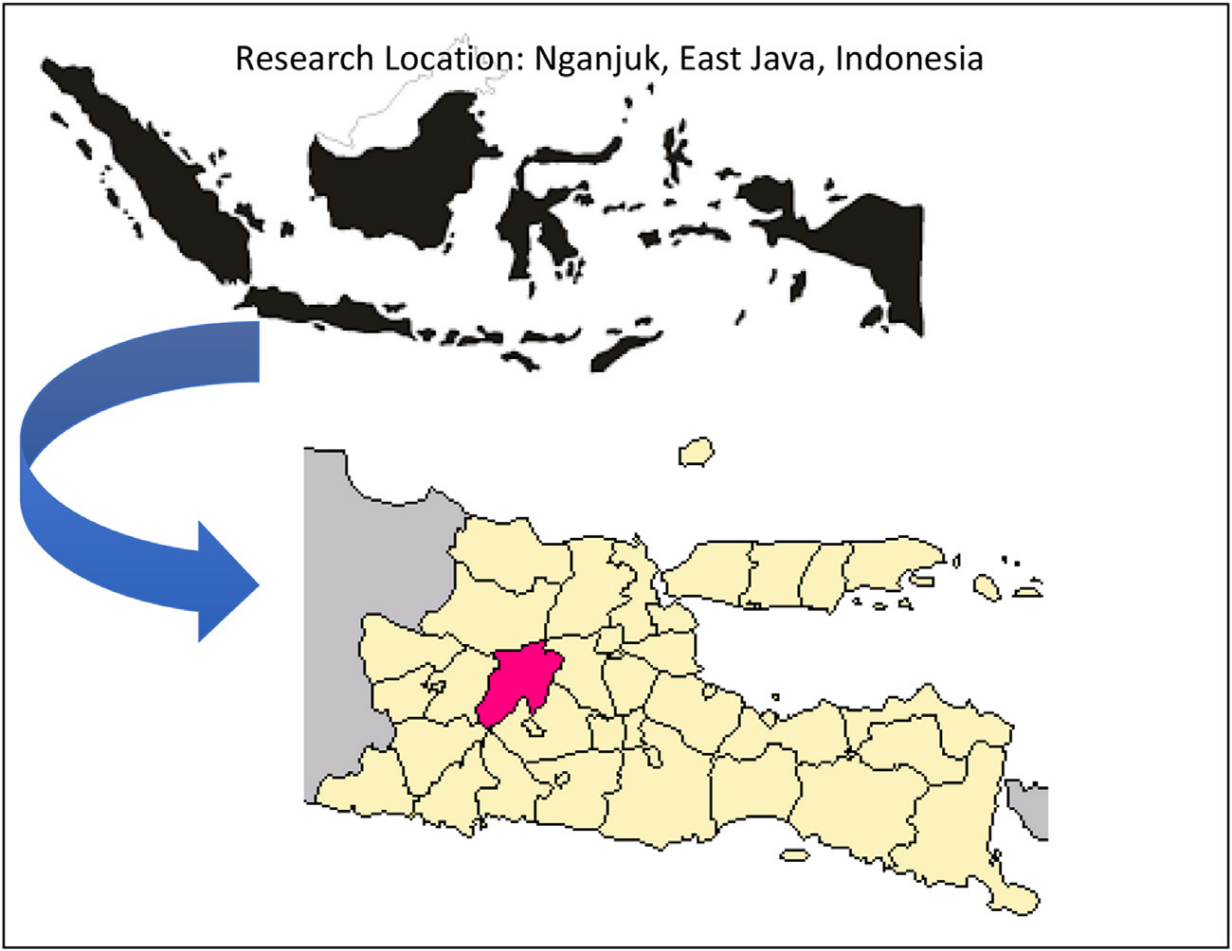
Location of Kabupaten Nganjuk, Propinsi Jawa Timur, Indonesia

This paper utiliized a case study based on occurrences that occured in the Indonesian regions to investigate the issue of group lending. The study was conducted in Indonesia's East Java Province's Nganjuk Regency [8]. Fig. 2 depicts the geographical mapping of the district's area [10].

**Fig. 3:**
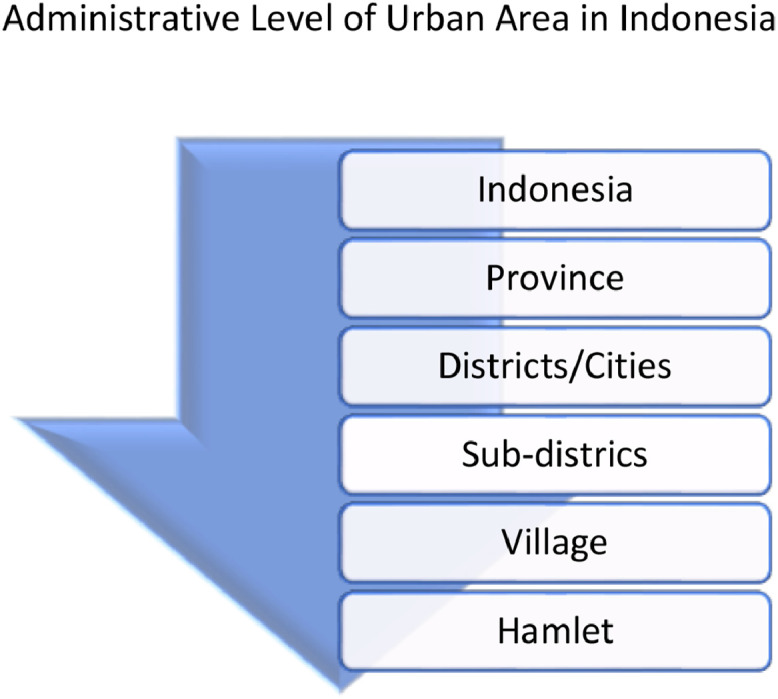
The Administrative level of government division areas in Indonesia geographically

One of the study's pillars concerned the geographical division of administration in rural areas. The division of government territory in Indonesia's geography consists of several administrative levels (Fig. 3). The central government, which has a national scope concerning Indonesia, is at the highest level. Next is the provincial level followed by districts or cities. Furthermore, each district/city is divided into several regions, known as sub-districts. The next level is the village, whereas the last level is the hamlet [11].

**Fig. 4:**
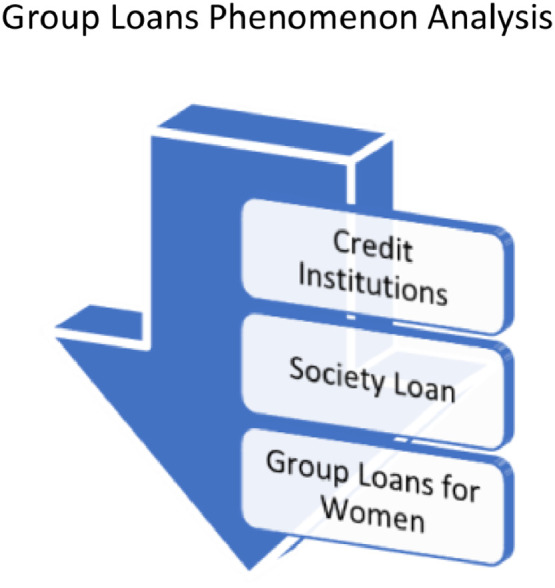
Analysis of the Group Loans Phenomenon in Women's Society

Phenomenon analysis was carried out through a stratified and inductive method. The initial stage began with a study of the existence of credit institutions. Then the study of credit occurred in the community (society loan) and finally narrowed down to Group Loans for women (Fig. 4).

### Credit institutions

The credit institution utilized in this study was microfinance institutions. At the stage of reviewing credit institutions, this study reviewed the understanding of the phenomenon of credit institutions in the global and Indonesian realms, especially credit institutions in the countryside. Knowledge of methods was studied in-depth and comprehensively through the study of literature.

The method of using microcredit facilities for rural women has been studied in various forms of research. Among the studies that have been conducted are such as the impact on the creation and opening of jobs [12] and overcoming financial barriers in households [13]. Various other studies related to the existence of this credit institution are also to get a more appropriate method. Reflections on which this study is based include social approaches to credit institutions [14], credit institutions in the face of global poverty [15], efficiency in credit institutions related to the social environment [16], and social and financial development [14]. In addition, other research are also related to the role of credit institutions in reducing poverty rates [17], the question of mission deviations by credit institutions through their performance [18], as well as the orientation of credit institutions and the determination of loan interest rates [19].

Other works include business competitions at credit agencies, the personal mission of field credit officers related to the credit institutions where they work [20] and the attachment of labor to credit agencies [21]. Other literature also includes studies from various countries related to microfinance: the improvement of microfinance facilities and social responsibility [22], and empirical analysis of the critical role of credit institutions in women's empowerment and poverty reduction [23].

In Indonesia, the credit distribution method is carried out, among others, through the Lembaga Perkreditan Desa (LPD), also known as the village credit institution [24]. LPD is a savings and loan business entity owned by the village and is one of the Village Owned Enterprises (BUM Desa).

BUM Desa is a village business institution managed by the community and the village government to strengthen the village economy and formed based on the needs and potential of the village. According to the Law of the Republic of Indonesia Number 32 of 2004 on Local Government, villages can establish business entities by the potential and needs of the village. BUM Desa has the primary function and purpose of raising community funds and distributing them by means of loans for productive activities [25]. Through credit institutions, distribution and financial turnover to the community regulates the regulations, being established by the government and Bank Indonesia [26]. In addition, credit for the people in Indonesia is also held in various forms. Other common forms are Cooperatives, People's Credit Bank (BPR), and Sharia People's Financing Bank (BPRS) [27].

### Society Loan

Literature and observation are the tools employed to investigate the phenomenon of community crediting. This study is supported by a scientific foundation built on the work of other scholars in a variety of ways such as a study related to the analysis of women's empowerment through microfinance institutions [28], loans on farmers' household loans in Vietnam [29], access to household credit which is influenced, among others, by the value of loans, the purpose of its application such as in agriculture, geographical and ethnic location [30], and financial literacy affecting financial behavior in society [31].

Researchers observed 20 sub-districts at the research location in relation to social loans committed in this study. Identified credit institutions are available in each sub-district at this time. This information is arranged into tables and saved in a database. PNPM, Mekar, Syariah, MBK, Komida, and Amartha are among the credit institutions located in the locations surveyed, according to field observations.

### Group loans for women

Studies at a later stage involved the phenomenon of Group Loans for Women. This phenomenon was carried out by means of literature study and observation methods. To benefit microcredit services, observations were carried out directly in the field, both to the micro-credit provider and to the community.

Other researchers have used a variety of methods in their scientific publications, including those connected to credit institutions that focus on lending money in groups and have a non-commercial character (D'espallier et al., 2013) [32]. Additional literature was published about providing skills for women tailored to interests and talents [33] and gender-related challenges in credit institutions [34]. In addition, studies related to the risk of problematic credit in group loans in rural areas [35], access to micro-loans, and the influence of couples in the utilization of loans [36], as well as the impact of financial lending to women related to the sustainability of credit institutions [37] were also analyzed.

### Problem analysis

The methods used in problem analysis were interviews and observations [38]. The interview involved various parties, especially microcredit service providers, indebted community groups, and finance institution customers. Observations were made simultaneously to rectify the problems revealed through interviews.

**Fig. 5:**
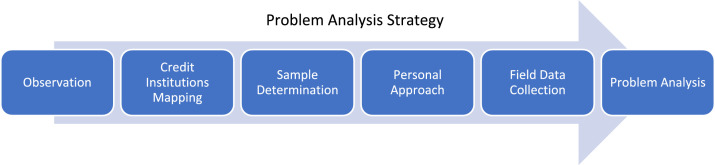
Problem analysis strategy on the phenomenon of group loans to women

This approach to society certainly faced challenges because the study concerned with the community's privacy, especially regarding loans to financial institutions. For this reason, particular practices and strategies were required. The technique applied to this study was compiled methods, as seen in Fig. 5. The stages of problem analysis strategy included Observation, Credit Institutions Mapping, Sample Determination, Personal Approach, Field Data Collection, and Problem Analysis.

### Observation

Observation was the initial stage of problem analysis. At this stage, direct observation was carried out in the field to obtain data on the existence of credit institutions.. Through observation, researchers collected data from credit institutions at the research site.

The observation target of this research was to get a phenomenon and list of credit institutions in all sub-districts at the research site. Collected data were recorded in electronic data. List of credit institutions based on the observation was stored in the repository.

### Credit institutions mapping

**Fig. 6:**
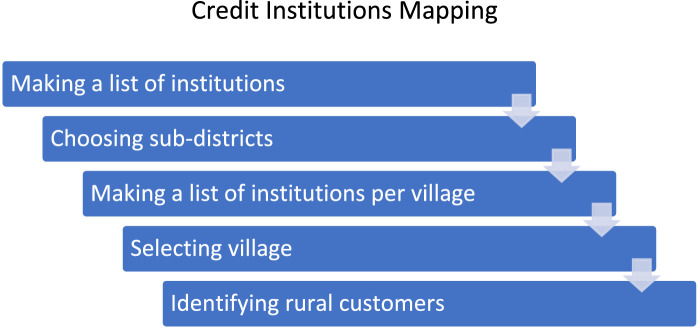
Stages of mapping credit institutions

The credit organization in question is a microfinance institution that used a Group Loan method to provide lending services. At the mapping stage of this credit institution, the method used was an interview with the credit institution at the research site. In this section, the following five steps were carried out: making a list of institutions, choosing sub-districts, making a list of institutions per village, selecting village, and identifying rural customers (Fig. 6).

In the early stages, researchers compiled a list of credit institutions at the research site based on sub-districts (data stored in the repository). Next, the sub-district was chosen. District selection was based on the number of lists of credit institutions. From the results of the existing list, the selected subdistrict was the Prambon sub-district. The next stage was to record the credit institution in Prambon Subdistrict (data stored in the repository). Through the list of credit institutions, villages were selected for research sample.In this study, the village chosen was Baleturi Village. After selecting a village, the most significant number of customer groups from each hamlet were further identified. Then, a list of customer groups and the number of members data were stored in the repository.

### Sample determination

The determination of the sample was based on rural customer data. The customer list, village, and hamlet were collected at the mapping stage. Next, the village and hamlet with the most significant number of customer groups were selected. The selected hamlet was the hamlet of Baleturi.

Interviews with financial institution employees were performed in addition to client groups. We also interviewed representatives from all of the financial institutions in the research area. The number of sample candidates interviewed were as many as six groups of people comprising customers of financial institutions representing customers of microfinance institutions. The number of individual respondents who were interviewed were 35, including representatives of credit institutions (data stored in the repository).

### Personal approach

**Fig. 7:**
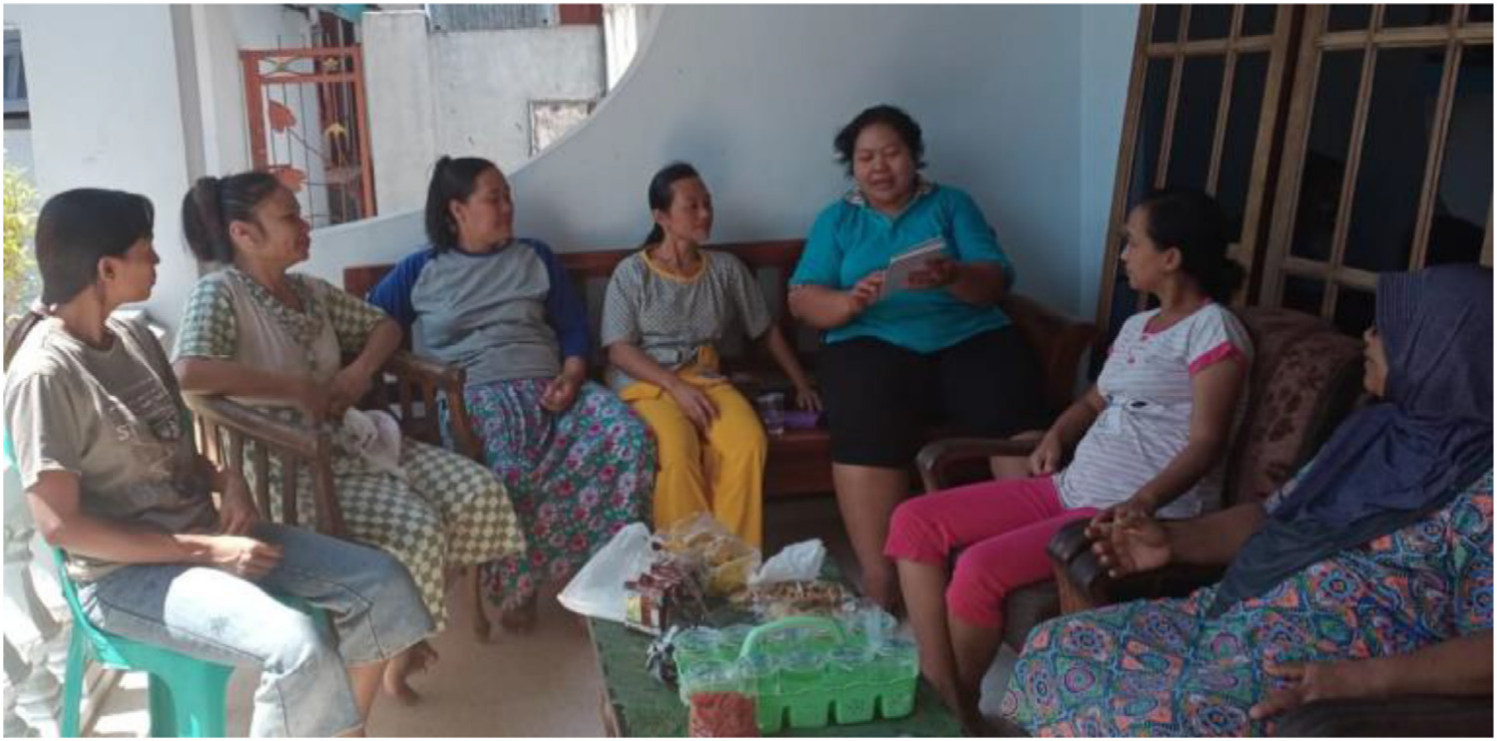
The process of approaching group loan customers by attending group meetings

The next step in the research was to map the situation of mothers who had become financial institution customers. We take a personal approach at this point. The tactic employed in this approach to society is to obtain data from credit agency personnel.. The purpose of extracting these data was to determine the customers who used Group Loan services. After getting customer data, the researchers met the group chairman and took a personal approach through formal and informal activities. Meetings between marketing and client groups, attendance at arisan (lottery club), studying, shopping, and seeing customers during a celebration event are all part of the method.. One of the documentation when approaching the community is shown in Fig. 7.

The next step was to establish a personal relationship between the researcher and candidate. The person being interviewed was a customer of the financial organisation. The researcher and candidate then agreed to meet again. Further meetings were held after deciding on the forum's schedule and the purpose of the visit through an interview. The interview took place at the house of each of the parties being interviewed.

### Field data collection

The next stage was field data collection. Field data collection was conducted through an interview or discussion to deepen the material to the customer group. Discussions were held between the researcher and the chairman or member of the group. The interview took place between April 3 and July 30, 2020.

Interviews were arranged in a structured format. Each interview result was recorded and documented in an electronic file. Files of interview results were reported in table format through worksheets—the results of this interview were stored in the repository.

### Problem analysis

**Fig. 8:**
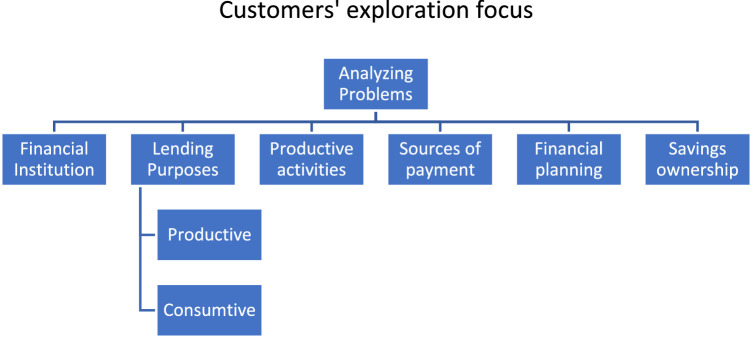
Customers' exploration focus

Problem analysis was more focused on looking at the customer side in groups. At this stage, the study of problems was explored by using credit institutions, lending purposes, productive activities, sources of payment, financial planning, and savings ownership (Fig. 8). Consumers' exploration was carried out through the structure of questions given to interviewed consumers.

The first area to be investigated is the financial institutions that consumers employ for the purpose of borrowing monies, whether for productive or consumptive purposes. They also interviewed customers about their productive activities, such as the sources of funds for consumer credit payments. Another aspect is connected to consumer financial planning, as well as determining whether they have saves funds in the form of savings.

The results of field interviews with consumers were recorded and documented. These field data were used as materials for problem analysis, and interview data became the basis for problem analysis—field data supported by observations as well. Field data were stored in the repository.

The results of field interviews were used to analyze the problem. Based on the problem analysis, it was found that there were problems that occurred to consumers. The respondents were using more than one financial institution. Most of the respondents used funds for consumptive activities. They paid debts owed to them by other financial institutions or from their husband's earnings.

Meanwhile, most respondents also revealed that they did not have productive jobs. In terms of payments, respondents were generally sourced from credit of other institutions. In short, they do not have savings, and they typically never participate in women's empowerment activities.

### Solution design

**Fig. 9:**
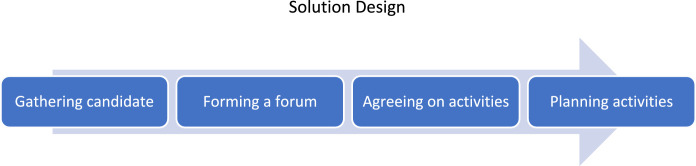
Solution design of non-performing loan payments related to the Group Loan system

Solution design was the stage for designing alternative solutions to the problem of non-performing loan payments related to the Group Loan system. The following four steps were carried out at this stage: gathering candidates, forming a forum, agreeing on activities and planning activities (Fig. 9).

Fig. 10.

**Fig. 10:**
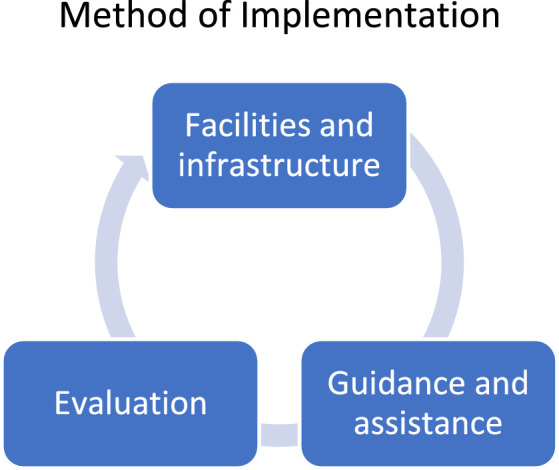
Methods of implementing women's empowerment activities in anticipating group debt problems

At this Solution Design stage, the first step was Gathering Candidates who had problems with the Group Loans. Researchers approach customers who identified as having issues related to Group Loans. Then, the researcher offered prospective members to join and participate in an association. This association is known locally as Paguyuban. Paguyuban is a forum for cadres within the community itself to participate in productive activities from a financial perspective.

After getting candidates to join, the next step was to form the management of the community. At this stage, the members shared tasks. Each member was assigned a job according to their respective abilities. This division of functions also formed a climate of responsibility for members to achieve organisational goals. Then, the next step was to agree with fellow members about the activities to be carried out. After deciding the type of activity, the members designed the activities to be carried out. In designing the action, several factors were considered in the design of this activity, including the location of the movement, facilities and infrastructure, and the need for an activity budget—data related to the activity profile stored in the repository.

### Implementation

During the implementation of activities, at least three activities were carried out. The three movements offered facilities and infrastructure, guidance and assistance, and evaluation and were carried out on an ongoing basis. Provision of facilities and infrastructure was carried out by cadres and researchers, adhering to the scheduled plans.

Furthermore, guidance and assistance were carried out so that community activity ran smoothly. This guidance and assistance included conducting briefings for cadres, organizing strategy and debriefing. Organization strategy covered the areas of management, production, marketing, and finance. The debriefing for these cadres included business development and optimization through cooperation, family economic management, and saving from the resulting income.

At the evaluation stage, the scope of the evaluation included business and members. Assessment on the business side was related to management, production, marketing, and finance. Meanwhile, it is related to savings, loan conditions, family financial management, and members' business impact from the member side. Data related to the evaluation results were stored in the repository.

### Method validation

The data related to the method carried out in this study were stored in the repository namely Mendeley Data [1]. Data of this research were available online. The data stored included records of a list of credit institutions in Nganjuk Regency, a list of credit institutions in Prambon District, mapping of customer groups by hamlet, interview data with both customers and officers from credit institutions, resumes of interviews with credit institutions, and resumes of interviews with individual customers.

## Conclusion

This study has analyzed the Group Loans and Women's Empowerment approach method. The discussion of the study includes phenomena, problems, and methods of overcoming issues related to Group Loans and Women's Empowerment. The phenomenon of the study comes from scientific research and case study. The problem is based on a case study that occurs in Nganjuk, East Java, Indonesia. Meanwhile, the problem-solving method is carried out in stages, starting from Phenomenon Analysis, Problem Analysis, Solution Design, and Implementation.

The method used in this research applies to a group of mothers who are trapped in a cycle of group loans. Through this method, they have formed a community, and through this community, they can carry out productive activities. The productive activities provide an alternative for mothers to play an active role and carry out activities that can help them fill their activities with financially positive activities and become an alternative for carrying out Women's Empowerment activities that entangle Group Loans.

This study can be one of the research solutions to mobilize Women's Empowerment in the community, significantly minimizing the risk of indebtedness to mothers in rural areas. Besides, of course, further research is needed to examine other alternatives to Women's Empowerment related to the Group Loans.

## Declaration of Competing Interest

None.

## Appendix Supplementary materials

Image, application 1
