# Supplementary material for: Methods for studying group loans, join responsibility, and women's empowerment
Source: MethodsX. 2022 Jun 2;9:101749. doi: 10.1016/j.mex.2022.101749 (PMC9192796; doi:10.1016/j.mex.2022.101749)
Supplement: Supplementary file 1 [file mmc1.pdf]

Sari Resources SA0202152-X

CERTIFICATE OF PROOFREADING/EDITING

This is to acknowledge that the article entitled

Methods for Studying Group Loans, Join Responsibility, and Women'sEmpowerment

Authored by

Efa Wahyu Prastyaningtyas
Sri Umi Mintarti Widjaja
Hari Wahyono
Endang Sri Andayani
Jack Febrian Rusdi

has been proofread/edited and is deemed

satisfactory by

…………………………………………………….

Dr. Zanariah Jano (PhD)

B.Ed (TESL), Univ of Winnipeg, Canada/ IELTS Trainer

Chief Consultant
